# Exploring the Physiological Pathways Connecting Adverse Childhood Experiences to Later Life Mortality Risk

**DOI:** 10.1093/geroni/igaf122.3010

**Published:** 2025-12-31

**Authors:** Taylor Brown

**Affiliations:** West Virginia University, Morgantown, West Virginia, United States

## Abstract

Adverse childhood experiences (ACEs) impact health and development well into adulthood, but less clear are the precise mechanisms. Thus, the current study utilized a sample of 1,198 participants (Mean age = 55;) from the Midlife Development in the U.S. (MIDUS) study to test whether retrospective reports of ACEs led to higher allostatic load (AL) scores in adulthood, resulting in an increased risk of death. An ACEs count score ranging from 0 (no adversity) to 20 (greater adversity) was created (physical and emotional abuse by family; low socioeconomic status). AL score (0 = lowest; 1 = highest) was constructed using 24 biomarkers from 7 different physiological systems measured from 2005-09 (para/sympathetic systems, HPA axis, cardiovascular, lipid/glucose metabolism, inflammation). Death status and survival time was recorded from 1995-96 when ACEs were retrospectively reported, through the censor date of December 2021(deceased = 217). Cox proportional hazards models were estimated in a structural equation modeling framework to assess indirect effects of ACEs on mortality risk via AL (controlling for age, sex, race, education, marital status). Higher ACEs predicted a significantly greater level of AL (B = 0.67; p < .05) and higher AL predicted an increased risk of death (HR = 1.46; p < .05). A significant mediated effect (IE = 0.03; TOT = 0.30; p < .05) suggested that ACEs predicted an increased risk of death due to accumulated physiological damage. Findings elucidate how early life events can lead to a cascade of physiological changes that persist into adulthood and ultimately impact longevity.

